# ADAMTS4 Reduction Contributes to Extracellular Matrix Deposition and Impaired Myogenesis in the Skeletal Muscle of Cigarette Smoke-Exposed Mice

**DOI:** 10.3390/biomedicines13020474

**Published:** 2025-02-14

**Authors:** Danyang Li, Yuqiang Pei, Long Liang, Zihan Wang, Xiaoyan Gai, Yongchang Sun

**Affiliations:** Department of Respiratory and Critical Care Medicine, Research Center for Chronic Airway Diseases, Peking University Third Hospital, Peking University Health Science Center, Beijing 100191, China; ydl1751257325@126.com (D.L.); peiyuqiang666@163.com (Y.P.); Logan1733@163.com (L.L.); wzh1996111@163.com (Z.W.)

**Keywords:** chronic obstructive pulmonary disease, COPD, cigarette smoke, ADAMTS4, extracellular matrix

## Abstract

**Background:** The extracellular matrix (ECM) plays a critical role in the proper regeneration of skeletal muscle. ECM remodeling has been reported in the skeletal muscle of chronic obstructive pulmonary disease (COPD), while the mechanisms remain poorly understood. **Methods:** In this study, we examined the dynamic interplay between ECM components and ECM enzymes in COPD skeletal muscle and cigarette smoke (CS) extract-treated C2C12 cells. C2C12 cells were further used to evaluate the role of a disintegrin and metalloproteinase with thrombospondin motif 4 (ADAMTS4) in ECM remodeling and myogenesis. **Results:** Chronic CS exposure induced the development of COPD and comorbid sarcopenia in C57BL/6J mice. Muscle fibrosis was observed in the gastrocnemius muscle of CS-exposed mice, accompanied by an upregulation of protein expression but a downregulation of mRNA levels of fibronectin and versican. We found that the discrepancy of mRNA and protein expression was attributed to the aberrant secretion of some ECM enzymes belonging to matrix metalloproteinases and ADAMTS proteases, especially ADAMTS4. CS exposure reduced ADAMTS4 expression in gastrocnemius muscles and C2C12 cells, and *Adamts4* knockdown induced fibronectin and versican accumulation and impeded myogenic process. **Conclusions:** Considering that recent studies have indicated an impaired skeletal muscle regeneration in COPD, we suggested that the restrained production of ADAMTS4 in response to CS could be involved in the damaged muscle regeneration through regulating skeletal muscle ECM in COPD. Targeting ECM enzymes may benefit the rehabilitation of COPD-related sarcopenia.

## 1. Introduction

Chronic obstructive pulmonary disease (COPD) is a common and preventable lung disease characterized by persistent airway limitation and chronic respiratory symptoms [1]. Many patients presenting with COPD are affected by other comorbidities, such as sarcopenia [2]. Sarcopenia is defined as a progressive decrease in muscle mass, strength, as well as physical performance [3]. Accordingly, 15.5–34% patients with COPD suffer from sarcopenia, which results in poorer exercise tolerance, a lower quality of life, as well as a higher hospitalization rate and mortality risk [4,5]. Smoking is a major risk factor both for COPD and sarcopenia [1,6]. With regard to the mechanisms involved in COPD-related sarcopenia, anabolic/catabolic imbalance, inflammation, hypoxia, oxidative stress, mitochondrial dysfunction, slow-to-fast fiber type shift and muscle denervation are well-documented [7,8]. However, little is known about the extracellular matrix changes in COPD.

Typically, the skeletal muscle extracellular matrix (ECM) can be divided into three morphologically distinct layers: the epimysium is a dense connective tissue surrounding the whole muscle; the perimysium extends from the epimysium and separates the entire muscle into individual muscle fascicles; the endomysium is a sophisticated membrane enclosing each muscle fiber [9]. ECM accounts for 1–10% of the skeletal muscle and consists of multiple components, including collagens, fibronectin, laminin, glycoproteins, proteoglycans and glycosaminoglycans [9,10]. So far, there have been a few studies that have shown changes in the skeletal muscle ECM of patients with COPD. A slight but pronounced fibrotic material accumulation was observed in the vastus lateralis muscle of patients with COPD [11]. Furthermore, altered protein expression of vastus lateralis muscle ECM components in COPD was confirmed using enzyme-linked immunosorbent assay [12]. Transcriptional perturbation in ECM-related genes was also noted in the quadricep muscles of patients with COPD through weighted gene go-expression network analysis [13]. Nevertheless, how its happens or what it results in remains obscure.

Generally speaking, ECM molecules not only provide the structural supports of muscle, but also participate in the process of muscle development, repair, regeneration and adaptive changes [9,14]. The remodeling of the ECM could influence the proliferation and differentiation of muscle stem cells (MuSCs), also known as satellite cells, in the skeletal muscle [9,14]. As an example, aberrant signals from aged MuSC niche caused by ECM changes have been shown to hinder the regenerative potential of skeletal muscle [15]. Further analysis indicated that elevated SPARC-related modular calcium-binding protein 2 (Smoc2) in the ECM impaired stem cell functionality through integrin beta-1/mitogen-activated protein kinase signaling in aged skeletal muscle [15]. However, even more impressively, collagen has been shown to accumulate in the muscle endomysium and perimysium with aging, and muscle stem cells have converted to fibroblasts under an aged ECM environment, which could further aggravate skeletal muscle fibrosis and impair their regenerative ability [16,17,18]. Interestingly, although several ECM components, such as COL1A1 and Smoc2, have been seen to accumulate in the ECM, their mRNA expression was downregulated or unchanged [15,16]. A similar phenomenon has been observed in patients with COPD [12]. These results suggest that post-transcriptional factors may exist in the dynamic regulation of ECM.

ECM enzymes play a crucial role in the remodeling and regulation of the ECM. The coordination between matrix metalloproteinases (MMPs) and tissue inhibitors of metalloproteinases (TIMPs) is regarded as the main regulator of ECM degrading [19]. The a disintegrin and metalloproteinase with thrombospondin motif (ADAMTS) superfamily is another group of ECM enzymes that primarily degrade various proteoglycans and glycosaminoglycans and participate in ECM remodeling-related diseases, such as articular cartilage diseases [20]. Previous evidence has indicated that several members belonging to MMPs and TIMPs were decreased in aged skeletal muscles, indicating an impaired ability to cleave ECM molecules [16]. Whether the enzyme deficiency also presents in COPD remains to be investigated. Moreover, MMPs and ADAMTSs have been demonstrated to be involved in the regulation of the migration, differentiation and regeneration of skeletal muscle cells [19,21,22]. For example, some members of ADAMTS proteases, such as ADAMTS1, ADAMTS5 and ADAMTS15, have been shown to promote muscle stem cell activation and myotube fusion [21,22]. However, whether ADAMTSs are involved in skeletal muscle regeneration by interacting with the extracellular matrix in COPD still needs to be explored further.

In this study, we revealed the presence of ECM deposition in the skeletal muscle of cigarette smoke (CS)-exposed mice, accompanied by inconsistent mRNA and protein expression levels of certain ECM molecules. We suggested that this discrepancy was brought about by a reduction in ECM enzymes, with ADAMTS4 showing the greatest decrease. Furthermore, we demonstrated that CS exposure led to ADAMTS4 reduction, which in turn, caused the accumulation of ECM molecules and inhibited the activation, proliferation and differentiation of muscle cells. In conclusion, the disruption of the homeostasis between ECM and ECM enzymes could contribute to the impaired regenerative capacity of skeletal muscle, thus aggravating COPD-related sarcopenia.

## 2. Materials and Methods

### 2.1. Animal Model

Six-week-old specific pathogen-free C57BL/6J female mice were obtained from Cyagen Biosciences Inc. (Santa Clara, CA, USA) and housed at the Department of Laboratory Animal Science of the Peking University Health Science Center. Animals were fed in individually ventilated cages and kept under a 12 h light/dark condition at 24 ± 2 °C. The experiment group was subjected to passive exposure to cigarette smoke twice daily, six days a week, for a total duration of 24 weeks using an exposure chamber system to develop the COPD mouse model. Each CS exposure session lasted 120 min, using 20 Marlboro cigarettes (tar: 10 mg, nicotine: 0.8 mg, CO: 11 mg). The control mice were exposed to room air during this session. After 24 weeks, the body weights were recorded, and the muscle force of the mice was assessed using Grip Strength Meter (Ubibiolab, Beijing, China) as previously described [23]. All animal procedures were approved by the Institutional Animal Care and Use Committee of the Peking University Health Science Center (approval number: BCAJ0269) and adhered to the “Guide for the Care and Use of Laboratory Animals: Eighth Edition (2011)”.

### 2.2. Cell Culture

The mouse C2C12 cell line was purchased from American Type Culture Collection (Manassas, VA, USA). C2C12 cells were cultured in Dulbecco’s modified Eagle medium (DMEM, Hyclone, South Logan, UT, USA) supplemented with 10% fetal bovine serum (Gibco, Waltham, MA, USA) and 1% penicillin-streptomycin (Hyclone, South Logan, UT, USA) under 5% CO_2_ at 37 °C. For myotube differentiation, the culture medium was replaced with DMEM containing 2% horse serum (Hyclone, South Logan, UT, USA), when C2C12 cell confluence reached 80%. Then, C2C12 cells or myotubes were treated with CS extract (CSE) or small interfering RNA (siRNA) for 24–48 h.

### 2.3. Histology Analysis

The left lung and gastrocnemius muscle were removed and fixed with 4% paraformaldehyde and an environment-friendly GD muscle fixative solution (Servicebio, Wuhan, China), respectively. Then, the samples were paraffin-embedded, sectioned at 4 μm thickness and stained with hematoxylin and eosin (H&E). The destructive index (DI) and mean linear intercept (MLI) of lung sections were measured to evaluate the severity of emphysema, while the cross-section area (CSA) of gastrocnemius muscle was calculated to measure the degree of sarcopenia according to our previous studies [23,24]. In addition, the extent of skeletal muscle fibrosis was evaluated using Masson’s trichrome staining.

### 2.4. Immunochemistry

Paraffin-embedded gastrocnemius muscle sections were first deparaffinized and rehydrated, followed by the removal of endogenous peroxidase activity and microwave-assisted antigen retrieval. After blocking nonspecific binding sites with 10% goat serum, sections were incubated with the rabbit anti-ADAMTS4 antibody (11865-1-AP, Proteintech, Rosemont, IL, USA) at 37 °C for 2 h and horseradish peroxidase-conjugated goat anti-rabbit lgG (ZSGB-Bio, Beijing, China) at 4 °C for 30 min sequentially. Visualization was achieved using a DAB Detection System kit (ZSGB-Bio, Beijing, China). Images were obtained using a NanoZommer S210 C13239-01 digital slide scanner (Hamamatsu Photonics K.K., Hamamatsu, Japan), and the staining intensity was calculated using Fiji software (version 2.16.0, National institutes of Health, Bethesda, MD, USA).

### 2.5. Preparation of CSE

CSE was prepared as previously described [24]. In brief, the smoke of five full-burning cigarettes was drawn and gradually bubbled into 10 mL DMEM (Hyclone, South Logan, UT, USA). The extract was deemed to be 100% CSE when the absorbance at 320 nm was adjusted to around 4.0, and it was then passed through a 0.22 µm filter (Millipore, Sigma, Billerica, MA, USA) to eliminate particles and bacteria. Cell counting kit-8 (CCK-8) was used to measure the cytotoxicity of different concentrations of CSE on C2C12 cells and C2C12 myotubes.

### 2.6. Small Interfering RNA Transfection

The siRNA targeting mouse *Adamts4* and negative control siRNA were purchased from GenePharma (Suzhou, China). C2C12 cells were seeded into six-well culture plates and transfected with siRNA when they reached 60% confluence. A total of 250 μL Opti-MEM medium containing 5 μL siRNA (20 nmol/l) and 7.5 μL Lipofectamine^®^ RNAiMAX transfection reagent (Invitrogen, Waltham, MA, USA) was added to the culture medium, and the incubation was carried out for 24–48 h for different purposes. A CCK-8 assay was performed to evaluate the effect of ADAMTS4 on cell proliferation. To investigate the effect of *Adamts4* knockdown on C2C12 cell differentiation, the culture medium was switched to DMEM containing 2% horse serum 24 h after transfection, and cells were collected and tested after another 24–72 h. The si-*Adamts4* sequences used are as follows: sense: 5′-GGAGAUGUUGCUACUAGAATT-3′, anti-sense: 5′-UUCUAGUAGCAACAUCUCCTT-3′.

### 2.7. Polymerase Chain Reaction (PCR)

Total RNA was extracted with TRIzol reagent (Life, Waltham, MA, USA) or RNA fast200 Total RNA Extraction Kit (Fastagen, Shanghai, China) according to the manufacturer’s instructions. Reverse transcription of RNA to cDNA was performed using an Evo M-MLV RT Kit with gDNA Clean for qPCR II (Accurate Biology, Changsha, China) and quantitative real-time PCR was conducted on a Bio-RAD CFX96 Connect Real-Time System (Hercules, CS, USA) with a SYBR Green Premix Pro Taq HS qPCR Kit (Accurate Biology, Changsha, China). The primer sequences are listed in Table 1, and the 2^−ΔΔCt^ method was used to calculate the relative changes in target gene expression normalized to the reference gene, GAPDH.

### 2.8. Immunoblotting

The total protein of gastrocnemius muscle homogenate or C2C12 cells was extracted using RIPA lysis buffer containing protease inhibitors (Applygen, Beijing, China) and quantified using a bicinchoninic acid (BCA) protein assay kit (Applygen, Beijing, China). Then, 30 μg protein was separated by SDS-PAGE and transferred to polyvinylidene fluoride (PVDF) membranes with a 0.45 μm pore size (Millipore, Sigma, Billerica, MA, USA). After blocking with 5% nonfat dry milk powder at room temperature for 2 h, the PVDF membranes were incubated with the rabbit anti-FBXO32 antibody (bsm-54451R, Bioss, Peking, China), rabbit anti-TRIM63 antibody (55465-1-AP, Proteintech, Rosemont, IL, USA), rabbit anti-COL1A1 antibody (72026, Cell Signaling Technology, Danvers, MA, USA), rabbit anti-fibronectin antibody (ET1702-25, HUABIO, Hangzhou, China), rabbit anti-versican antibody (A19655, ABclonal, Wuhan, China) or rabbit anti-MyHC antibody (10799-1-AP, Proteintech, Rosemont, IL, USA) at 4 °C overnight and HRP-labeled goat anti-rabbit secondary antibody (ZSGB-Bio, Beijing, China) at room temperature for 1 h, sequentially. Blots were visualized using Immobilon Western Chemiluminescent HRP Substrate (Millipore, Billerica, MA, USA) with an automatic chemiluminescence image analysis system (Tanon, Shanghai, China).

### 2.9. Immunofluorescence

Cells were fixed in 4% paraformaldehyde for 15 min and incubated with 5% bovine serum albumin for 1 h to block non-specific sites. For intracellular staining, cells were permeabilized with 0.3% Triton X-100 for 15 min after fixation. Then, cells were stained with the rabbit anti-fibronectin antibody (ET1702-25, HUABIO, Hangzhou, China), rabbit anti-versican antibody (A19655, ABclonal, Wuhan, China) or mouse anti-Myosin Heavy Chain antibody (MAB4470, R&D systems, Minneapolis, MN, USA) at 4 °C overnight. After washing with PBS three times, cells were probed with Alexa Fluro 488-conjugated goat anti-rabbit IgG or Alexa Fluro 594-conjugated goat-anti mouse IgG at room temperature for 1 h. DAPI (4,6-diamino-2-phenylindole, Beyotime, Shanghai, China) was applied to stain the cell nuclei. Images were obtained with a laser scanning confocal microscope and processed using Zen 3.3 software (Carl Zeiss, Jena, Germany).

### 2.10. Statistical Analysis

Statistical analyses were processed with GraphPad Prism 8 software (GraphPad Software, La Jolla, CA, USA). Data were presented as the mean ± standard error of the mean (SEM). Following the normality test of the data, an unpaired Student’s *t* test or Mann–Whitney U test was used to compare the two groups, and a one-way ANOVA test followed by Dunnett’s multiple comparisons test was chosen to compare multiple groups. Results were considered as significant for *p* < 0.05.

## 3. Results

### 3.1. Chronic CS Exposure Induced the Development of Sarcopenia in C57BL/6J Mice

**Figure 1:**
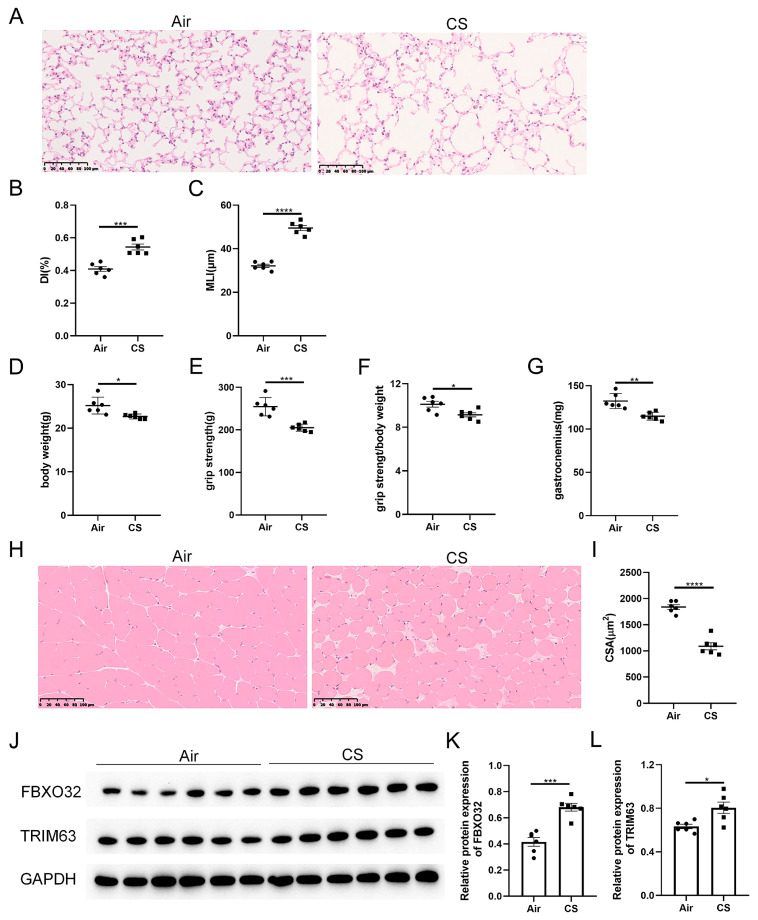
Chronic exposure to cigarette smoke (CS) caused emphysema and sarcopenia in C57BL/6J mice. (**A**) Representative hematoxylin–eosin staining images of lung parenchyma of air-exposed and CS-exposed mice. The severity of emphysema was assessed using destructive index (DI) (**B**) and mean linear intercept (MLI) (**C**). The body weight (**D**), grip strength (**E**), the ratio of grip strength to body weight (**F**) and gastrocnemius muscle weight (**G**) of two groups of mice were measured. (**H**) Representative hematoxylin–eosin staining images of gastrocnemius muscles. (**I**) The cross-section area (CSA) of gastrocnemius muscles was calculated. (**J**–**L**) Immunoblot and densitometric analysis of FBXO32 and TRIM63. Scale bar = 100 μm. *n* = 6. * *p* < 0.05, ** *p* < 0.01, *** *p* < 0.001, **** *p* < 0.0001.

As shown in our previous studies [24], prolonged exposure to CS results in the destruction of lung parenchyma, a classical feature of COPD, as evidenced by increased DI and MLI indexes in CS-exposed mice (Figure 1A–C). Unexpectedly, these CS-exposed mice exhibited a lower body weight, decreased grip strength, as well as a reduced ratio of grip strength to body weight compared with air-exposed mice (Figure 1D–F). Meanwhile, decreased weight and cross-section area of gastrocnemius muscle were observed in CS-exposed mice (Figure 1G–I). In addition, the skeletal muscle protein degradation markers, F-box protein 32 (FBXO32, also known as atrogin-1) and tripartite motif containing 63 (TRIM63, also known as MuRF-1), were increased in response to CS exposure (Figure 1J–L). These results indicated that 24-week CS exposure induced mice to develop COPD and sarcopenia.

### 3.2. Chronic CS Exposure Induced Skeletal Muscle Extracellular Matrix Deposition in C57BL/6J Mice

**Figure 2:**
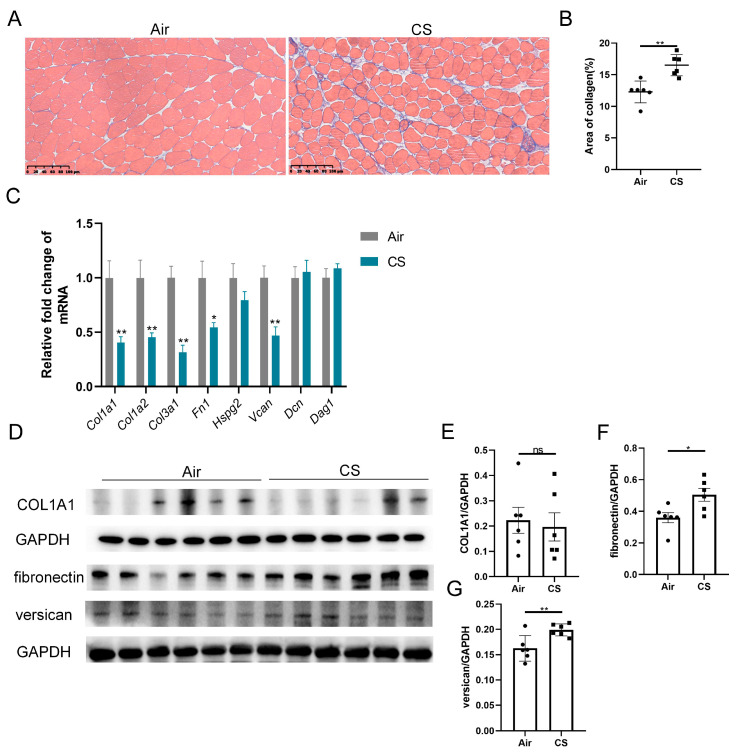
Chronic exposure to cigarette smoke (CS) induced skeletal muscle extracellular matrix remodeling in C57BL/6J mice. (**A**) Representative Masson’s trichrome staining images of gastrocnemius muscles of air-exposed and CS-exposed mice, scale bar = 100 μm. (**B**) Quantification of collagen in gastrocnemius muscles. (**C**) The mRNA expression of some extracellular matrix components, including *Col1a1*, *Col1a2*, *Col3a1*, *Fn1*, *Hspg2*, *V0/V1 Vcan*, *Dcn* and *Dag1*, in gastrocnemius muscle homogenates. (**D**–**G**) Immunoblot and densitometric analysis of COL1A1, fibronectin and versican in gastrocnemius muscle homogenates. *n* = 6. * *p* < 0.05, ** *p* < 0.01, ns: no significance.

Previous studies have reported ECM remodeling in the skeletal muscle of patients with COPD, with mild yet significant fatty cell replacement and fibrosis observed through myopathological examination [11]. Similarly, we noticed a looser skeletal muscle structure and increased collagen fiber content in CS-exposed mice compared with air-exposed mice (Figure 2A,B). We next examined the transcription levels of a series of ECM components, including collagens (*Col1a1*, *Col1a2*, *Col3a1*), fibronectin (*Fn1*), heparan sulfate proteoglycan 2 (*Hspg2*), versican V0/V1 splice variant (*V0/V1 Vcan)*, decorin (*Dcn*) and dystroglycan 1 (*Dag1*). Interestingly, three genes of collagens, *Fn1* and V0/V1 *Vcan* mRNA were significantly downregulated in response to CS exposure (Figure 2C). However, CS-exposed mice showed an upregulated protein expression of fibronectin and versican compared with air-exposed mice, with no significant changes in the COL1A1 protein level (Figure 2D–G). These findings revealed an inconsistency in the mRNA and protein expression of ECM components.

### 3.3. The Expression of ADAMTS4 Was Decreased in the Gastrocnemius Muscle of CS-Exposed Mice

**Figure 3:**
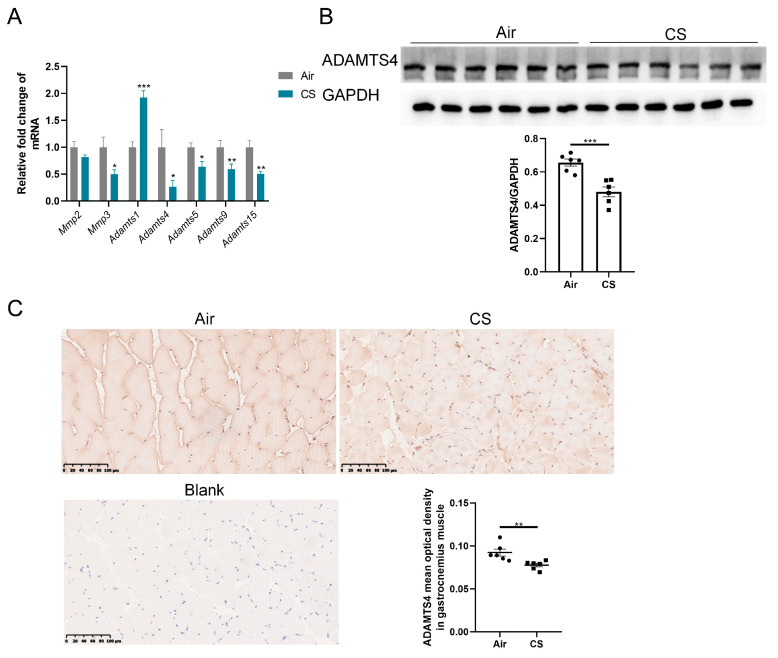
The expression of ADAMTS4 was decreased in mouse gastrocnemius muscle in response to cigarette smoke (CS) exposure. (**A**) The mRNA expression levels of *Mmp2*, *Mmp3*, *Adamts1*, *Adamts4*, *Adamts5*, *Adamts9* and *Adamts15* were assessed in the gastrocnemius muscle of air-exposed and CS-exposed mice. (**B**) The protein level of ADAMTS4 in gastrocnemius muscle homogenate was measured by immunoblotting. (**C**) Representative images of ADAMTS4 in the gastrocnemius muscle of air-exposed and CS-exposed mice and corresponding analysis, scale bar = 100 μm. *n* = 6. * *p* < 0.05, ** *p* < 0.01, *** *p* < 0.001.

The discrepancy between mRNA and protein expression of ECM-related proteins could be induced by several reasons, such as post-translational modifications, reduced protein degradation and increased translational efficiency. Given the crucial role of ECM enzymes in the degradation of ECM proteins, we first investigated the expression of some enzymes belonging to the MMP and ADAMTS family, which have been reported to exist and function in the skeletal muscle of mice. MMPs are well-documented for their involvement in the degradation of collagens, whereas ADAMTSs primarily interact with fibronectin and versican [19,22,25]. The mRNA expression of *Mmp3* and several ADAMTS family members (*Adamts4*, *Adamts5*, *Adamts9* and *Adamts15*) was reduced in gastrocnemius muscle homogenate of CS-exposed mice compared with air-exposed mice, with a particularly notable decrease in *Adamts4* mRNA (Figure 3A). However, no significant alteration in *Mmp2* mRNA expression was observed in the gastrocnemius muscle of CS-exposed mice compared with air-exposed mice, and *Adamts1* showed a marked upregulation (Figure 3A). Furthermore, we performed immunoblotting and immunochemical analyses to confirm that CS exposure decreased ADAMTS4 protein levels in mouse skeletal muscle fibers (Figure 3B,C). Considering the role of ADAMTS4 in the degradation of fibronectin and versican [25,26], we contended that ADAMTS4 might play a key role in ECM deposition in COPD mice.

### 3.4. CSE Induced ADAMTS4 Reduction and Extracellular Matrix Deposition in C2C12 Cells

**Figure 4:**
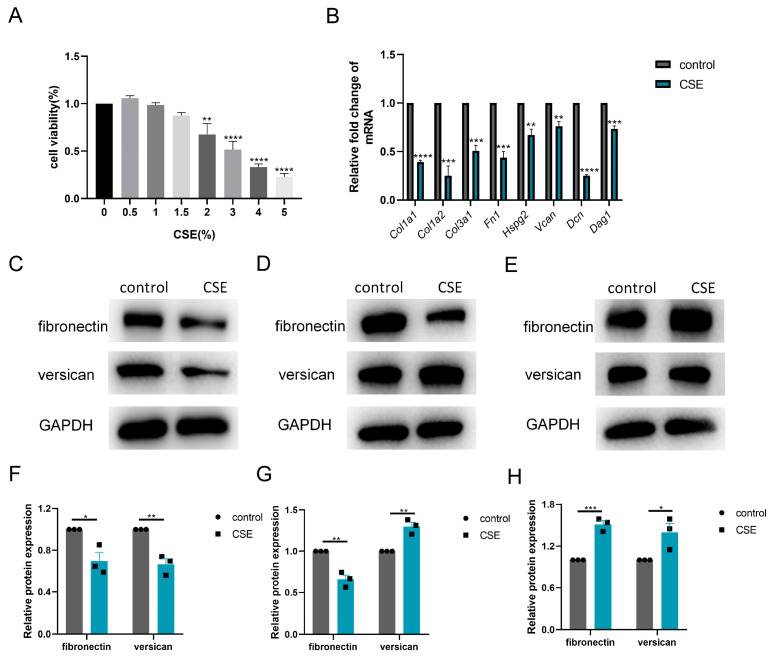
Cigarette smoke (CS) exposure induced changes in the production of extracellular matrix components in C2C12 cells. (**A**) CCK-8 assay was performed to evaluate the cytotoxicity of different concentrations of CS extract (CSE) to C2C12 cells, *n* = 4. (**B**) C2C12 cells were treated with 1.5% CSE for 24 h. The transcription levels of *Col1a1*, *Col1a2*, *Col3a1*, *Fn1*, *Hspg2*, *V0/V1 Vcan*, *Dcn* and *Dag1* were assessed, *n* = 4. Immunoblotting was used to evaluate the expression of fibronectin and versican in C2C12 cell after 18 (**C**,**F**), 24 (**D**,**G**) or 48 (**E**,**H**) hours of CSE addition, *n* = 3. * *p* < 0.05, ** *p* < 0.01, *** *p* < 0.001, **** *p* < 0.0001.

**Figure 5:**
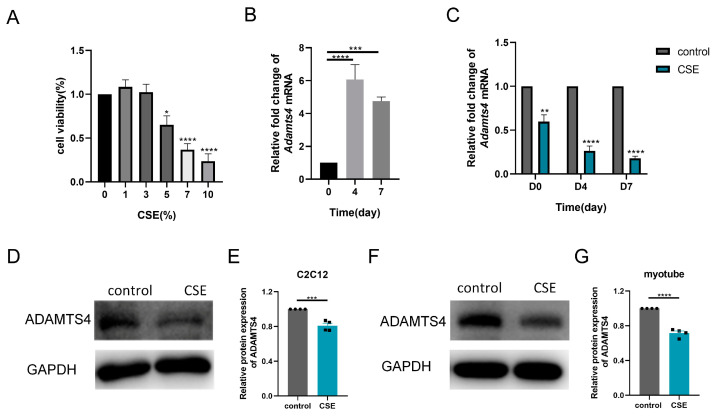
Cigarette smoke (CS) exposure decreased the expression of ADAMTS4 in C2C12 cells and the differentiating myotubes. (**A**) CCK-8 assay was performed to evaluate the cytotoxicity of different concentrations of CS extract (CSE) to C2C12 myotubes, *n* = 4. (**B**) The transcription level of *Adamts4* was measured in the C2C12 myotubes differentiated on day 0, day 4 and day 7, *n* = 5. (**C**) C2C12 cells and C2C12 myotubes differentiated on day 3 and day 6 were treated with CSE for 24 h. The expression of *Adamts4* mRNA was assessed, *n* = 4. Immunoblotting was used to evaluate the protein level of ADAMTS4 in C2C12 cells (**D**,**E**) and C2C12 myotubes differentiated on day 7 (**F**,**G**) with or without CSE addition, *n* = 4. * *p* < 0.05, ** *p* < 0.01, *** *p* < 0.001, **** *p* < 0.0001.

Thereafter, we investigated the subtle relationship between ECM components and ECM enzymes in vitro. According to the CCK-8 assay, 1.5% CSE was used to treat C2C12 cells, while 3% CSE was applied to C2C12 myotubes (Figure 4A and Figure 5A). We showed that CSE treatment decreased the mRNA expression of multiple ECM components, including *Col1a1*, *Col1a2*, *Col3a1*, *Fn1*, *Hspg2*, *Vcan*, *Dcn* and *Dag1*, in C2C12 cells (Figure 4B). However, a reduction in versican and fibronectin protein levels was only observed in the early stage of CSE addition, with gradual deposition of versican and fibronectin after treatment with CSE longer than 24 h (Figure 4C–H). Similar to other members of the ADAMTS family [22], the mRNA expression of *Adamts4* was increased and then decreased during myotube differentiation, with the peak expression occurring on day 4. (Figure 5B). Nevertheless, qPCR results showed that the expression of MMPs (*Mmp2*, *Mmp3*) and ADAMTSs (*Adamts1*, *Adamts4*, *Adamts5*, *Adamts9*, *Adamts15*) mRNA was dramatically downregulated after CSE treatment, both in C2C12 cells and in C2C12 myotubes differentiated on day 4 and day 7 (Figure 5C and Figure S1). The reduction of ADAMTS4 in response to CSE addition in C2C12 cells and myotubes was also confirmed by immunoblotting (Figure 5D–G).

### 3.5. Knockdown of ADAMTS4 Interfered with Fibronectin and Versican Secretion in C2C12 Cells

**Figure 6:**
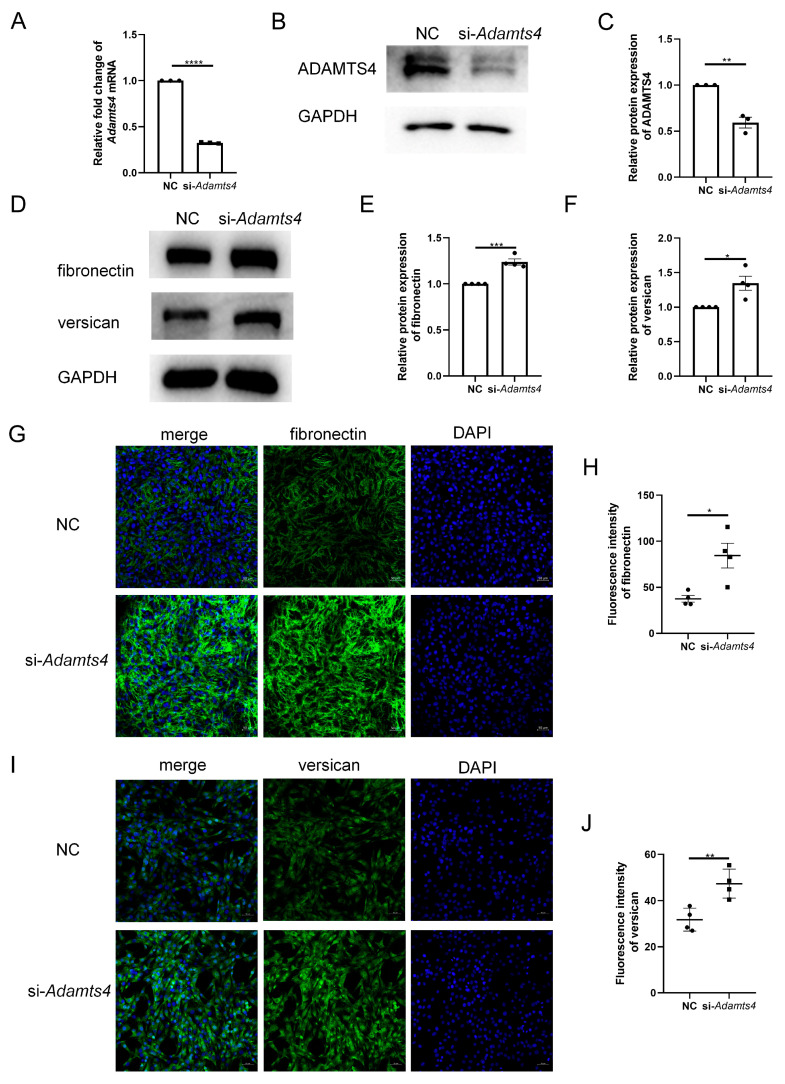
*Adamts4* knockdown resulted in fibronectin and versican accumulation in C2C12 cells. (**A**–**C**) The transfection efficiency of small interfering RNA targeting mouse *Adamts4* (si-*Adamts4*) was evaluated at the mRNA and protein levels, *n* = 3. (**D**–**F**) C2C12 cells were transfected with si-*Adamts4* or negative control siRNA (NC) for 48 h, and the protein levels of fibronectin and versican were measured, *n* = 4. (**G**–**J**) Representative immunofluorescence images of fibronectin and versican and corresponding quantification of mean fluorescence intensity. Scale bar = 50 μm, *n* = 4. * *p* < 0.05, ** *p* < 0.01, *** *p* < 0.001, **** *p* < 0.0001.

To investigate whether ADAMTS4 directly interferes with ECM remodeling in C2C12 cells, we constructed an siRNA targeting *Adamts4*, which effectively reduced the mRNA and protein expression of ADAMTS4 (Figure 6A–C). Transfection with si-*Adamts4* led to fibronectin and versican accumulation in C2C12 cells, which was demonstrated by immunoblotting and immunofluorescence analyses (Figure 6D–J).

### 3.6. ADAMTS4 Participated in the Activation, Proliferation and Differentiation of C2C12 Cells

**Figure 7:**
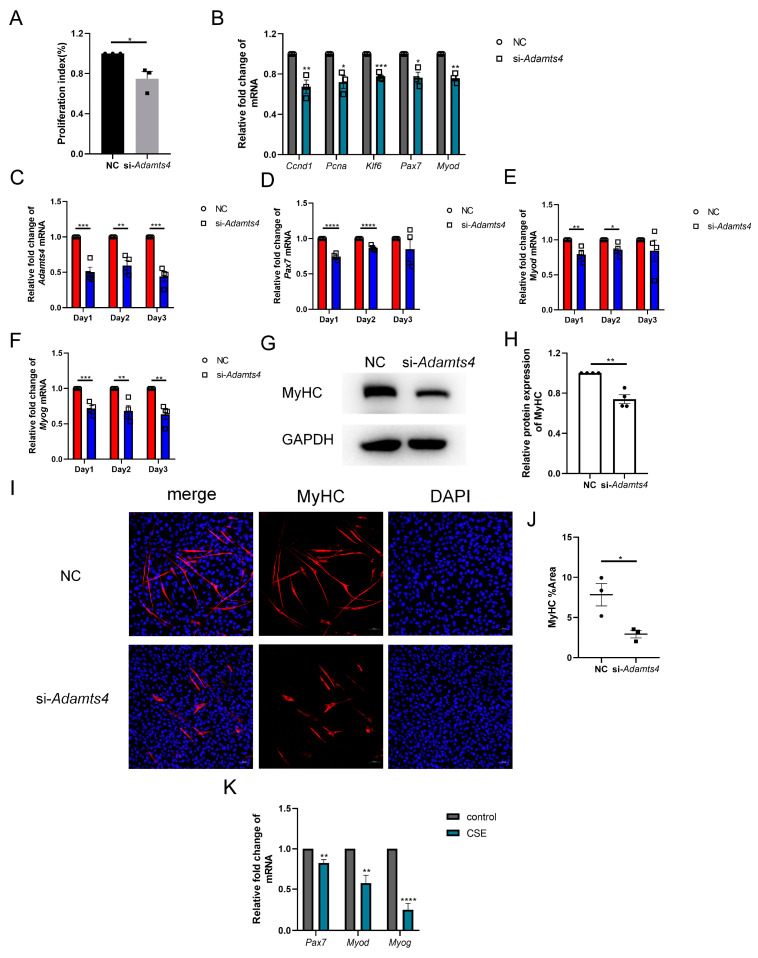
*Adamts4* knockdown interfered the myogenesis of C2C12 cells. (**A**) The proliferation index of C2C12 cells was measured by CCK-8 assay, *n* = 3. (**B**) The mRNA levels of *Ccnd1*, *Pcna*, *Klf6*, *Pax7* and *Myod* were detected in C2C12 cell transfected with si-*Adamts4* or negative control siRNA (NC) for 24 h, *n* = 3. (**C**–**F**) C2C12 cells were transfected with si-*Adamts4* or NC for 24 h, and cultured in the differentiating medium for another one to three days. The mRNA levels of *Adamts4*, *Pax7*, *Myod* and *Myog* were measured, *n* = 4. (**G**,**H**) C2C12 cells were transfected with si-*Adamts4* or NC for 24 h, and cultured in the differentiating medium for 48 h. The protein expression of MyHC was evaluated by immunoblotting, *n* = 4. (**I**,**J**) Representative immunofluorescence images of MyHC in the differentiating C2C12 cells and the quantification of the area of MyHC-positive cells. Scale bar = 50 μm, *n* = 3. (**K**) C2C12 cells were treated with cigarette smoke extract (CSE) for 24 h, and the transcription levels of *Pax7*, *Myod* and *Myog* was assessed, *n* = 4. * *p* < 0.05, ** *p* < 0.01, *** *p* < 0.001, **** *p* < 0.0001.

Interestingly, we observed that C2C12 cells transfected with si-*Adamts4* for 24 h showed a lower cell confluence, which was further confirmed using the CCK-8 assay (Figure 7A). The qPCR results also indicated that the transcription levels of cell proliferation markers, such as *Ccnd1*, *Pcna* and *Klf6*, were reduced in C2C12 cells treated with si-*Adamts4* (Figure 7B). In addition, muscle stem cell-specific activation and early differentiation markers, *Pax7* and *Myod* mRNA, were decreased in *Adamts4* knockdown C2C12 cells (Figure 7B). Furthermore, we cultured the *Adamts4* knockdown C2C12 cells in differentiation medium, and monitored the mRNA expression levels of *Adamts4*, *Pax7*, *Myod*, as well as the late differentiation marker, *Myog*, for three consecutive days. We found that the expression of all four genes was decreased in the differentiating C2C12 cells transfected with si-*Adamts4* over three days, with *Pax7* and *Myod* reaching significance only on days 1 and 2 (Figure 7C–F). Moreover, the important marker for myotube formation, myosin heavy chain (MyHC), showed a decreasing trend in the differentiating *Adamts4* knockdown cells compared to the untreated cells (Figure 7G–J). To sum up, knockdown of *Adamts4* impaired the activation, proliferation and differentiation of C2C12 cells.

Deficient myogenic ability of skeletal muscle satellite cells has been reported in COPD patients and mice [27,28,29,30]. We also observed the reduction of *Pax7*, *Myod* and *Myog* mRNA levels in CSE-treated C2C12 cells (Figure 7K). In addition, recent studies have demonstrated that ECM deposition could damage the myogenic capacity of satellite cells [15,18]. Therefore, we suggested that the decreased expression of ADAMTSs, especially ADAMTS4, could contribute to the compromised regenerative potential of skeletal muscle stem cells by promoting fibronectin and versican deposition in CS-exposed mice, thereby leading to COPD-related sarcopenia.

## 4. Discussion

In this study, we developed a COPD mouse model through exposure to CS for 24 weeks. As expected, the chronic CS-exposed mice displayed an emphysematous phenotype combined with skeletal muscle wasting. The gastrocnemius muscle showed enlarged interstitial spaces and mild increased collagen area among muscle fibers, suggesting that intramuscular fat accumulation and fibrosis occurred in CS-exposed mice. This phenomenon has also been observed in the skeletal muscle of patients with COPD [11], aging individuals [16,31], as well as other diseases with muscle atrophy, such as type 2 diabetes mellitus [32]. Further analysis indicated that the content of fibronectin and versican was increased in the gastrocnemius muscle of CS-exposed mice compared to that in air-exposed mice, whereas the protein level of COL1A1 was unchanged. Likewise, a reduction in collagen type Ⅰ and an increase in fibronectin expression have been reported in the vastus lateralis muscle of patients with COPD [12]. In contrast, in aged mice, the expression of COL1A1 was found to be increased [16], while the fibronectin level was decreased [33]. This inconsistency highlighted that, beyond aging, additional factors may influence the ECM remodeling process in COPD. Among them, CS exposure could be an important reason. Smokers with COPD displayed a lower collagen type Ⅰ expression and higher fibronectin level compared with non-smoker controls in freshly isolated airway smooth cells, and in vitro culture with CSE addition exhibited a same trend [34]. From another perspective, the reduction in COL1A1 protein level in CS-exposed mice implied that other collagen proteins, such as COL1A2 and COL3A1, could contribute to the increased collagen fraction area.

Remarkably, although fibronectin and versican were elevated at the protein level, their mRNA expression was reduced. This finding prompted us to investigate post-transcriptional factors that regulate the protein levels of ECM molecules. We found that the mRNA expression of several ECM enzymes, including *Mmp3*, *Adamts4*, *Adamts5*, *Adamts9* and *Adamts15*, was reduced in the gastrocnemius muscle of CS-exposed mice compared with air-exposed mice. Since ADAMTS4 showed the largest decrease among them, we next focused on this molecule. The protein expression of ADAMTS4 was reduced in CS-exposed mice, in parallel with the gene results.

Furthermore, we examined the dynamic interplay of ECM components and ECM enzymes in vitro. CSE treatment significantly reduced the mRNA expression of ECM molecules in C2C12 cells, whereas the accumulation of versican and fibronectin proteins was observed after 24 h and 48 h, respectively. Similar to CS-exposed mice, the CSE-treated C2C12 cells demonstrated a pronounced decline in the transcription levels of ECM enzymes, including MMP2, MMP3, ADAMTS1, ADAMTS4, ADAMTS5, ADAMTS9 and ADAMTS15. Therefore, it is possible that the decrease in enzyme production prolonged the half-life of ECM molecules, leading to their accumulation. In addition, no matter if CSE was added in the middle or late stages of myotube differentiation, the mRNA expression of these enzymes was reduced. The protein expression of ADAMTS4 was also lower in CSE-treated C2C12 cells and C2C12 myotubes than in untreated controls. Hence, exposure to CS affects the entire developmental process of muscle cells, from stem cells to fully differentiated mature fibers, leading to a reduction in ECM enzyme expression. Above all, we proved that CS exposure induced the reduction of enzymes, especially ADAMTS4, and ECM deposition in muscle cells in vivo and in vitro.

ADAMTS4 is a zinc metalloproteinase that has been extensively studied in various ECM remodeling-related diseases, such as osteoarthritis [35,36], atherosclerosis [37,38], aortic aneurysms and dissections [26], and so on. ADAMTS4-mediated aggrecan degradation in articular cartilage is widely recognized as a primary etiological factor in the pathogenesis of osteoarthritis, while its cleavage of both aggrecan and versican plays a crucial role in the instability of atherosclerotic plaques [35,37]. The proteolysis of versican by ADAMTS4 also contributes to fetal membrane rupture at parturition [39], as well as the development of thoracic aortic aneurysms and dissections [26]. Besides the degradation of proteoglycans, ADAMTS4 has the ability to cleave extra domain A-fibronectin [25]. In the current study, we found that *Adamts4* knockdown significantly increased fibronectin and versican levels in C2C12 cells, suggesting that their cleavage in muscle cells was repressed.

Appropriate levels of ECM components are conducive for muscle repair and regeneration, and conversely, deviations from these levels can be detrimental. For example, fibronectin was demonstrated to be an indispensable component for MuSC expansion and myogenic differentiation [40,41]. Loss of fibronectin in the aged MuSC niche diminished the adhesive property of MuSCs, thereby contributing to MuSC aging and impairing the regenerative capacity of skeletal muscle in aged mice [33]. In contrast, excess fibronectin inhibited myoblast fusion [42]. Moreover, versican showed a protective effect for cardiac repair by promoting cardiomyocyte proliferation [43]. However, versican processing was required for myotube formation, and high levels of versican did harm for this process [22,44,45]. Considering the ECM fibrosis in C2C12 cells transfected with si-*Adamts4*, we further examined whether *Adamts4* knockdown also affected the myogenic ability of muscle stem cells.

Previous studies have indicated a role of ADAMTS4 in embryonic myogenesis [46]. *ADAMTS4* rs41270041 polymorphism is associated with muscle function deficit in childhood acute lymphoblastic leukemia [47]. Additionally, other molecules belonging to the ADAMTS protease family or ADAMTS-like proteins (ADAMTSL) have been shown to play a role in muscle development and regeneration. Overexpression of ADAMTS1 in macrophages suppressed Notch1 signaling, thereby triggering satellite cell activation and facilitating muscle regeneration after injury [21]. The versicanase catalytic activity of ADAMTS5 and ADAMTS15 was prerequisite for muscle fiber formation [22]. Moreover, myoblast-derived ADAMTSL2 potentiated canonical WNT signaling and promoted myoblast differentiation and muscle regeneration [48,49]. During the myogenic process, several master transcription factors are involved. Following injury, MuSCs are activated and Pax7 expression is increased, leading to their proliferation into myogenic differentiation protein (MyoD)-positive myoblasts. These myoblasts then differentiate into myotubes, a process characterized by the upregulation of myogenin, and downregulation of both Pax7 and MyoD [50]. In the present study, we observed reduced activation and proliferation markers in C2C12 cells 24 h after *Adamts4* siRNA treatment. During the differentiation process, the mRNA levels of *Pax7*, *Myod* and *Myog* were decreased by *Adamts4* knockdown. Myotube formation was also inhibited, as assessed by immunoblotting and immunofluorescence analyses of MyHC. These results indicated an important role of ADAMTS4 in C2C12 myogenesis, which could be related to their processing of fibronectin and versican.

Recently, reduced regenerative capacity has been demonstrated in patients and mice with COPD. Although the number of Pax7^+^ satellite cells did not differ between patients with COPD and controls [27,51,52], Pax7^+^Myf5^−^ quiescent satellite cells showed a reduction in patients with COPD compared with control subjects [53]. The proportion of Pax7^+^Myf5^+^-activated satellite cells was reported to be significantly increased in the vastus lateralis muscle of COPD patients compared with controls [53], and the percentage of central nuclei-positive fibers, which represent newly formed muscle cells, was elevated in patients with COPD without muscle atrophy compared to controls, indicating an attempt to repair the injured muscle [27,52]. Increased expression of MyoD and myogenin in muscle homogenates of COPD patients and mice also supports this speculation [27,54], although decreased or unchanged levels of these markers have been observed in different studies [53,55]. However, in patients with COPD and muscle atrophy (mid-thigh cross-sectional area <70 cm^2^), the counts of central nuclei-positive fibers were decreased [27,52]. Upon muscle injury, CS-exposed mice exhibited lower Pax7^+^ centralized nuclei within tibialis anterior muscle compared with wild-type mice. In addition, COPD satellite cells isolated and cultured in vitro displayed a deficiency in myotube fusion and MyHC production [27,28,29,30]. All of the above results suggested a decline in skeletal stem cell function in COPD. Likewise, as shown in the previous results, CSE treatment impaired the myogenic process in C2C12 cells. Therefore, it is more likely that CS exposure damaged skeletal muscle regeneration through regulating ADAMTS4 in COPD.

Our study has some limitations. First, we lack the animal experiments to explore the protective effect of ADAMTS4 on muscle regeneration in COPD, such as by exogenous injection of ADAMTS4 into the skeletal muscle of CS-exposed mice. Furthermore, the upregulated signaling of ADAMTS4 in response to CS exposure was unknown. In addition, whether other ADAMTS members (such as ADAMTS5, ADAMTS9 and ADAMTS15) are also involved in muscle regeneration through modulating ECM components in COPD requires further investigation. The increased ADAMTS1 production in the gastrocnemius muscle of CS-exposed mice may more or less compensate for the effect of these reduced ADAMTS molecules, as the members of the ADAMTS family share similar functions. Last but not least, these findings need to be validated in patients with COPD. Transcriptomic and proteomic studies of skeletal muscle from COPD patients with sarcopenia could provide a more comprehensive understanding of ECM-associated protein alterations, explore common underlying mechanisms and help identify targets for enzyme-based therapies.

## 5. Conclusions

In this study, we identified an unbalanced interaction between ECM and ECM enzymes in the skeletal muscle of COPD. Among these, ADAMTS4 was a key ECM enzyme whose reduction led to fibronectin and versican accumulation and damaged the myogenic ability of skeletal muscle stem cells. Targeting ECM enzymes may represent a promising therapeutic strategy to enhance the regenerative capacity of skeletal muscle, hence alleviating sarcopenia associated with smoking-related COPD.

## Figures and Tables

**Table 1:** Primer sequences for qPCR.

Target	Forward Sequence (5′-3′)	Reverse Sequence (5′-3′)
*Col1a1*	GCTCCTCTTAGGGGCCACT	CCACGTCTCACCATTGGGG
*Col1a2*	GGTGAGCCTGGTCAAACGG	ACTGTGTCCTTTCACGCCTTT
*Col3a1*	ACGTAGATGAATTGGGATGCAG	GGGTTGGGGCAGTCTAGTG
*Fn1*	GCTCAGCAAATCGTGCAGC	CTAGGTAGGTCCGTTCCCACT
*Hspg2*	TGGAGCCCGAATACAGGAAGA	AGATCCGTCCGCATTCCCT
*V0/V1 Vcan*	ACCAAGGAGAAGTTCGAGCA	CTTCCCAGGTAGCCAAATCA
*Dcn*	TCTTGGGCTGGACCATTTGAA	CATCGGTAGGGGCACATAGA
*Dag1*	CTTGAGGCGTCCATGCACT	GGCAATTAAATCCGTTGGAATGC
*Mmp2*	CAAGTTCCCCGGCGATGTC	TTCTGGTCAAGGTCACCTGTC
*Mmp3*	ACATGGAGACTTTGTCCCTTTTG	TTGGCTGAGTGGTAGAGTCCC
*Adamts1*	AAGGAAGAAGCGATTTGTGTCC	CCACCGAGAACAGGGTTAGA
*Adamts4*	ATGGCCTCAATCCATCCCAG	AAGCAGGGTTGGAATCTTTGC
*Adamts5*	GGAGCGAGGCCATTTACAAC	CGTAGACAAGGTAGCCCACTTT
*Adamts9*	GACTTGTGGGCAAGGTAAGG	TCAGTCTCGGGGATGTAATCTG
*Adamts15*	GCTCATCTGCCGAGCCAAT	CAGCCAGCCTTGATGCACTT
*Ccnd1*	GCGTACCCTGACACCAATCTC	CTCCTCTTCGCACTTCTGCTC
*Pcna*	TTTGAGGCACGCCTGATCC	GGAGACGTGAGACGAGTCCAT
*Klf6*	GTTTCTGCTCGGACTCCTGAT	TTCCTGGAAGATGCTACACATTG
*Pax7*	CGATTAGCCGAGTGCTCAGA	GGAGGTCGGGTTCTGATTCC
*Myod*	ATAGACTTGACAGGCCCCGA	GTAGGGAAGTGTGCGTGCT
*Myog*	AATGCACTGGAGTTCGGTCC	TTCGTCTGGGAAGGCAACAG
*Gapdh*	AGGTCGGTGTGAACGGATTTG	GGGGTCGTTGATGGCAACA

## Data Availability

The original contributions presented in this study are included in the article. Further inquiries can be directed to the corresponding authors.

## Supplementary Materials

The following supporting information can be downloaded at: https://www.mdpi.com/article/10.3390/biomedicines13020474/s1, Figure S1: Cigarette smoke extract (CSE) treatment decreased the expression of extracellular matrix-related enzymes in C2C12 cells and the differentiating myotubes.
