# Supplementary material for: ADAMTS4 Reduction Contributes to Extracellular Matrix Deposition and Impaired Myogenesis in the Skeletal Muscle of Cigarette Smoke-Exposed Mice
Source: Biomedicines. 2025 Feb 14;13(2):474. doi: 10.3390/biomedicines13020474 (PMC11853528; doi:10.3390/biomedicines13020474)
Supplement: Supplementary file 1 [file biomedicines-13-00474-s001.zip › biomedicines-3466552-supplementary.pdf]

Supplementary Materials

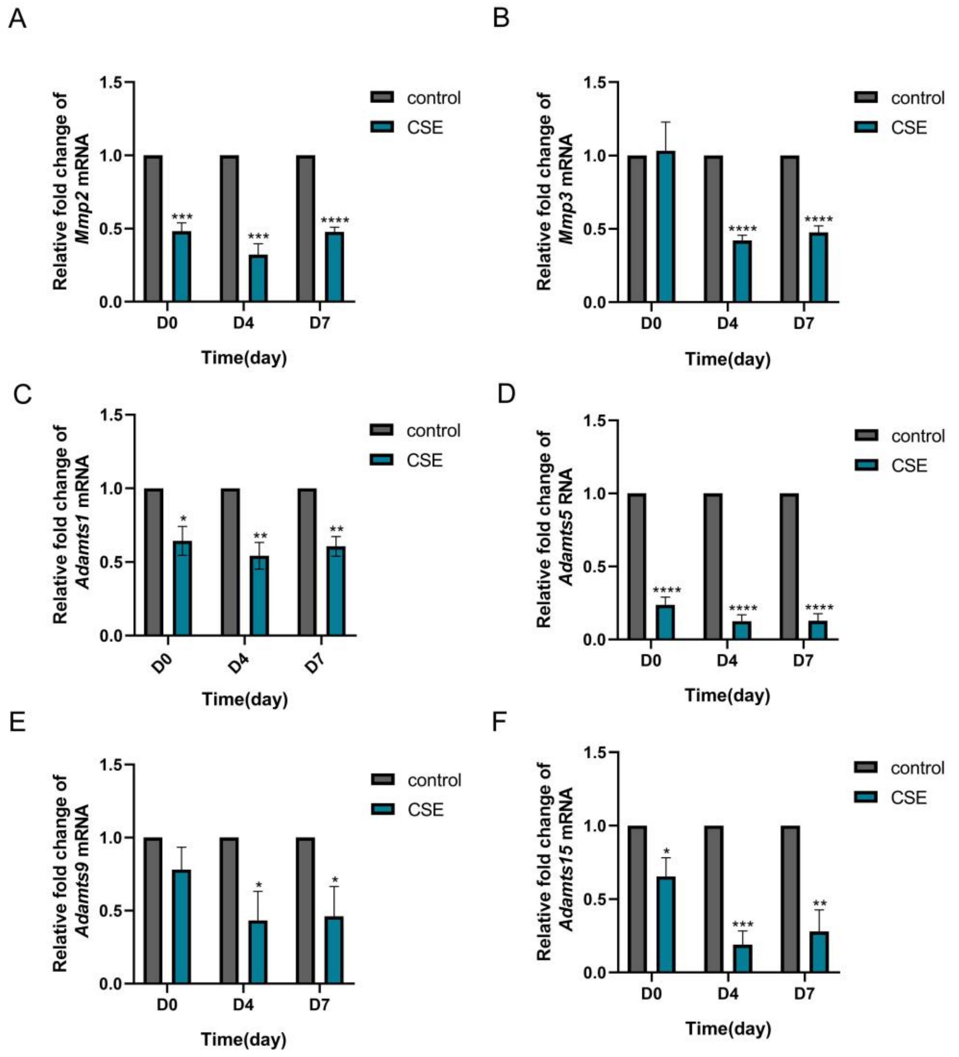

Figure S1: Cigarette smoke extract (CSE) treatment decreased the expression of extracellular matrix-related enzymes in C2C12 cells and the differentiating myotubes. C2C12 cells and C2C12 myotubes differentiated on day 3 and day 6 were treated with CSE for 24 h. The expression of *Mmp2* (A), *Mmp3* (B), *Adamts1* (C), *Adamts5* (D), *Adamts9* (E), and *Adamts15* (F) mRNA was assessed, n = 4. * $p < 0.05$, ** $p < 0.01$, *** $p < 0.001$, **** $p < 0.0001$.
